# Large J expansion in ABJM theory revisited

**DOI:** 10.1140/epjc/s10052-014-3042-9

**Published:** 2014-09-11

**Authors:** H. Dimov, S. Mladenov, R. C. Rashkov

**Affiliations:** 1 Department of Physics, Sofia University, 5 J. Bourchier Blvd, 1164 Sofia, Bulgaria; 2 Institute for Theoretical Physics, Vienna University of Technology, Wiedner Hauptstr. 8-10, 1040 Vienna, Austria

## Abstract

Recently there has been progress in the computation of the anomalous dimensions of gauge theory operators at strong coupling by making use of the AdS/CFT correspondence. On the string theory side they are given by dispersion relations in the semiclassical regime. We revisit the problem of a large-charge expansion of the dispersion relations for simple semiclassical strings in an $\mathrm{AdS}_4\times {\mathbb {CP}}^3$ background. We present the calculation of the corresponding anomalous dimensions of the gauge theory operators to an arbitrary order using three different methods. Although the results of the three methods look different, power series expansions show their consistency.

## Introduction

Currently the world-volume dynamics of branes is the most important player in the duality between gauge theories and strings (M-theory).

Last years there have appeared a large number of works focused on the understanding of the world-volume dynamics of multiple M2-branes and the near horizon limit of the effective geometry. This interest was inspired by the investigations of Bagger et al. [1–4] on theories having hidden structures of a Lie 3-algebra and which have an intimate relation to membrane dynamics. On other hand the progress in the AdS/CFT correspondence motivates one to look for a new class of conformal invariant, maximally supersymmetric field theories in $2+1$ dimensions describing the world-volume dynamics of coincident membranes in M-theory. Indeed, a dual pair of theories corresponding to the above picture was found [5, 6] and it triggered a large number of investigations in various directions. On one side the so-called Aharony–Bergman–Jafferis–Maldacena (ABJM) theory consist of $N$ membranes on $S^7/{\mathbb Z}_k$ and membranes in M-theory on $\mathrm{AdS}_4\times S^7/{\mathbb Z}_k$, or after reduction on an M-theory cycle to type IIA string theory, strings in an $\mathrm{AdS}_4\times {\mathbb {CP}}^3$ background. On the dual gauge theory side the theory is $\mathcal{N}=6$ superconformal Chern–Simons theory coupled with bifundamental matter (actually two Chern–Simons theories of level $k$ and $-k$, respectively, and each with gauge group $SU(N)$). The superstrings on $\mathrm{AdS}_4\times {\mathbb {CP}}^3$ as a coset were first studied in [7]^1^ opening the door for investigation of the integrable structures in the theory. Shortly after that it was noticed that the string supercoset model does not describe the entire dynamics of a type IIA superstring in $\mathrm{AdS}_4 \times {\mathbb {CP}}^3$, but only its subsector. The complete string dual of the $\mathcal{N}=6$ superconformal Chern–Simons theory, i.e. the complete type-IIA Green–Schwarz string action in $\mathrm{AdS}_4\times {\mathbb {CP}}^3$ superspace has been constructed in [9].

Being highly non-linear, the theory on both sides is hard to solve exactly. Thus, the semiclassical analysis appears to be the most appropriate available tool to answer many questions. The duality between the two theories suggests that the partition functions of string theory on $\mathrm{AdS}_4\times {\mathbb {CP}}^3$ and $\mathcal{N}=6$ superconformal Chern–Simons theory are equal. If one works on the string side, one can find the semiclassical string solutions and work out the string spectrum. According to the AdS/CFT correspondence, the dispersion relations on the string theory side are equal to the dimensions of the gauge theory operators. Therefore, one of the main ingredients necessary to check the holographic correspondence is the dimension of the gauge theory operators.

Although the issue of dispersion relations was addressed in some papers, see for instance^2^ [7–24], in this note we revisit the problem studying the large momentum expansion of folded string dispersion relations. The difficulty is that the conserved charges for corresponding semiclassical string solutions are typically represented in terms of elliptic integrals. The latter are difficult to invert, carefully separating leading, subleading etc. orders of the contributions. In this note we consider three methods for calculating the anomalous dimensions of the gauge theory operators by the AdS/CFT correspondence. The results agree, but they have quite different forms.

This paper is organized as follows. In Sect. 1 we give a very brief review of the basics of ABJM theory and review a simple folded string solution in the ${\mathbb {CP}}^3$ part of the geometry. Next we apply the iterative method for inverting the elliptic integrals to obtain the dispersion relations corresponding to the folded string solution. The second method used for calculation of the dispersion relations is the Picard–Fuchs equation. Finally we present the calculation of the anomalous dimensions to the first few orders using the Lambert function as advocated in [25] for the case of $\mathrm{AdS}_5\times S^5$.

### On ABJM theory in brief

The $\mathrm{AdS}_4/\mathrm{CFT}_3$ correspondence, or ABJM theory, is one of the rare candidates for the exact string/gauge theory correspondence. It is obtained starting from 11d M-theory analyzing the M2-brane dynamics. One starts with M2-brane solutions obtained considering the 11 dimensional supergravity action [6]

1.1 $$\begin{aligned} S&= \frac{1}{2\kappa _{11}^2}\int \mathrm{d}x^{11}\sqrt{-g}\left( R-\displaystyle \frac{1}{2\cdot 4!}F_{\mu \nu \rho \sigma }F^{\mu \nu \rho \sigma }\right) \nonumber \\&\quad -\frac{1}{12\kappa _{11}^2} \int C^{(3)}\wedge F^{(4)}\wedge F^{(4)}, \end{aligned}$$

where $\kappa _{11}^2=2^7\pi ^8 l_p^9$. The M2-brane solutions can be obtained from 11d SUGRA equations of motions,

1.2 $$\begin{aligned} R^\mu _\nu =\frac{1}{2}\left( \frac{1}{3!}F^{\mu \alpha \beta \gamma }F_{\nu \alpha \beta \gamma } -\frac{1}{3\cdot 4!}\delta ^\mu _\nu F_{\alpha \beta \rho \sigma }F^{\alpha \beta \rho \sigma }\right) \end{aligned}$$

and

1.3 $$\begin{aligned} \partial _{\sigma }(\sqrt{-g}F^{\sigma \mu \nu \xi })\!=\!\frac{1}{2\cdot (4!)^2}\epsilon ^{\mu \nu \xi \alpha _1\dots \alpha _8}F_{\alpha _1\dots \alpha _4}F_{\alpha _5\dots \alpha _8}. \end{aligned}$$

For the purpose of the AdS/CFT correspondence we are interested in the near horizon limit of the $\mathrm{AdS}_4\times S^7$ spacetime

1.4 $$\begin{aligned} \mathrm{d}s^2=\frac{R^2}{4}\mathrm{d}s^2_{\mathrm{AdS}_4}+R^2 \mathrm{d}s^2_{S^7}, \end{aligned}$$

where the $\mathrm{AdS}_4$ cycle supports $N'$ units of four-form flux

1.5 $$\begin{aligned} F^{(4)}=\frac{3R^3}{8}\epsilon _{\mathrm{AdS}_4}, \quad R=l_p(2^5 N'\pi ^2)^{\frac{1}{6}}. \end{aligned}$$

Let us consider the quotient $S^7/\mathbb Z_k$ where $\mathbb {Z}_k$ is acting as $z_i \rightarrow \mathrm{e}^{ i \frac{2 \pi }{ k} } z_i$. A convenient way to proceed is to write the metric on $S^7$ as

1.6 $$\begin{aligned} \mathrm{d}s^2_{S^7} = ( \mathrm{d} \varphi ' + \omega )^2 + \mathrm{d}s^2_{{\mathbb {CP}}^3}, \end{aligned}$$

where

1.7 $$\begin{aligned}&\mathrm{d}s^2_{{\mathbb {CP}}^3} = \frac{ \sum _i \mathrm{d} z_i \mathrm{d} \bar{z}_i}{r^2 } - \frac{ | \sum _i z_i \mathrm{d} \bar{z}_i |^2}{ r^4},\quad r^2 \equiv \sum _{i=1}^4 |z_i|^2, \nonumber \\&\mathrm{d} \varphi ' + \omega \!\equiv \! \frac{ i}{2 r^2 } ( z_i \mathrm{d} \bar{z}_i \!-\! \bar{z}_i \mathrm{d} z _i ), \quad \mathrm{d}\omega \!=\! J \!=\! { i} d \left( \frac{ z_i}{r}\right) d \left( \frac{ \bar{z}_i}{ r }\right) , \nonumber \\ \end{aligned}$$

and then perform the ${\mathbb Z}_k$ quotient identifying $\varphi '=\varphi /k$ with $\varphi \sim \varphi +2\pi$. Noticing that $J$ is proportional to the Kähler form on ${\mathbb {CP}}^3$ one can write the resulting metric as

1.8 $$\begin{aligned} \mathrm{d}s^2_{S^7/{\mathbb Z}_k} = \frac{ 1}{k^2 } ( \mathrm{d} \varphi + k \omega )^2 + \mathrm{d}s^2_{{\mathbb {CP}}^3}. \end{aligned}$$

The consistent quantization of the flux forces the condition $N'=kN$, where $N$ is the number of quanta of the flux on the quotient. In this way the spectrum of the resulting theory will be that of the initial $\mathrm{AdS}_4\times S^7$ projected onto the ${\mathbb Z}_k$ invariant sector. In this setup there is a natural definition of ’t Hooft coupling $\lambda \equiv N/k$ and the decoupling limit is $N,k\rightarrow \infty$ while $N/k$ is kept fixed [6].

On the gauge theory side it was conjectured that $N$ multiple M2-branes on ${\mathbb {C}}^4/{\mathbb {Z}}_k$ are described by $\mathcal {N} = 6$ supersymmetric Chern–Simons-matter theory with gauge group $U(N) \times U(N)$ and levels $k$ and $-k$, respectively.

The Chern–Simons part of the theory is constructed using a pair of chiral fields $A_i$ ($i = 1, 2$) in the bifundamental representation $(\mathbf {N},\bar{\mathbf {N}})$, and a pair $B_i$ in the anti-bifundamental representation $(\bar{\mathbf {N}},\mathbf {N})$. The theory is supplied with a $\mathcal {N} = 2$ superpotential

1.9 $$\begin{aligned} W =\frac{4\pi }{k}\mathrm {Tr}(A_1B_1A_2B_2 - A_1B_2A_2B_1), \end{aligned}$$

where the scalar components of $(A_1,A_2,B^\dagger _1,B^\dagger _2)$ transform in the $\mathbf {4}$ of $SU(4)_R$ and the conjugate in the $\bar{\mathbf {4}}$. The $\mathcal {N} = 6$ supersymmetry is combined with an exact conformal symmetry organized in the $OSp(6|4)$ superconformal symmetry group.

**ABJM and strings on** $\mathrm{AdS}_4\times {\mathbb {CP}}^3$ One can now follow [6] to make a reduction to type IIA with the following final result:

1.10 $$\begin{aligned}&\mathrm{d}s^2_{\mathrm{string}} = \frac{ R^3}{ k} \left( \frac{ 1 }{ 4 } \mathrm{d}s^2_{\mathrm{AdS}_4} + \mathrm{d}s^2_{{\mathbb {CP}}^3} \right) , \end{aligned}$$

1.11 $$\begin{aligned}&\mathrm{e}^{2 \phi } = \frac{ R^3 }{ k^3 } \sim \frac{ N^{1/2} }{ k^{5/2} }= \frac{ 1 }{ N^2 } \left( \frac{N }{ k } \right) ^{5/2}, \end{aligned}$$

1.12 $$\begin{aligned}&F_{4} = \frac{ 3 }{ 8 } { R^3} \epsilon _4 , \quad F_2 = k \mathrm{d} \omega = k J. \end{aligned}$$

We end up then with an $\mathrm{AdS}_4 \times {\mathbb {CP}}^3$ compactification of type IIA string theory with $N$ units of $F_4$ flux on $\mathrm{AdS}_4$ and $k$ units of $F_2$ flux on the ${\mathbb {CP}}^1 \subset {\mathbb {CP}}^3$ 2-cycle.

The radius of the curvature in string units is $R^2_{\mathrm{str}} = \frac{ R^3}{ k} = 2^{5/2} \pi \sqrt{ \lambda }$. It is important to note that the type IIA approximation is valid in the regime where $k \ll N \ll k^5$.

To proceed, we will need the explicit form of the metric on $\mathrm{AdS}_4\times {\mathbb {CP}}^3$ in spherical coordinates. A convenient form of the metric on $\mathrm{AdS}_4\times {\mathbb {CP}}^3$ is [26]

1.13 $$\begin{aligned} \mathrm{d}s^2&= R^2\left\{ \dfrac{1}{4}[ -\mathrm {cosh}^2\rho \,\mathrm{d}t^2+\mathrm{d}\rho ^2+\mathrm {sinh}^2\rho \,\mathrm{d}\Omega _2^2]+\mathrm{d}\mu ^2 \right. \nonumber \\&\quad +\sin ^2\mu \left[ \mathrm{d}\alpha ^2+\dfrac{1}{4}\sin ^2\alpha (\sigma _1^2 +\sigma _2^2+\cos ^2\alpha \sigma _3^2) \right. \nonumber \\&\quad \left. \left. +\dfrac{1}{4}\cos ^2\mu (\mathrm{d}\chi +\sin ^2\mu \sigma _3)^2\right] \right\} . \end{aligned}$$

Here $R$ is the radius of the $\mathrm{AdS}_4$, and $\sigma _{1,2,3}$ are left-invariant 1-forms on an $S^3$, parameterized by $(\theta ,\phi ,\psi )$,

1.14 $$\begin{aligned}&\sigma _1=\cos \psi \,\mathrm{d}\theta +\sin \psi \sin \theta \,\mathrm{d}\phi , \nonumber \\&\sigma _2=\sin \psi \,\mathrm{d}\theta -\cos \psi \sin \theta \,\mathrm{d}\phi , \\&\sigma _3=\mathrm{d}\psi +\cos \theta \,\mathrm{d}\phi . \nonumber \end{aligned}$$

The range of the coordinates is

$$\begin{aligned}&0\le \mu ,\,\alpha \le \dfrac{\pi }{2},\,\,0\le \theta \le \pi ,\,\, 0\le \phi \le 2\pi ,\\&\quad 0\le \chi ,\,\psi \le 4\pi . \end{aligned}$$

### Review of the simplest semiclassical string solutions in $\mathrm{AdS}_4\times {\mathbb {CP}}^3$ background

As we noticed above the Chern–Simons terms are with opposite sign levels, namely $k$ and $-k$, while the superpotential has an $SU(2) \times SU(2)$ global symmetry acting on the pairs $A_i$ and $B_i$, respectively. In the near horizon limit with $k$ and $N$ satisfying $k \ll N \ll k^5$, the field theory is dual to IIA superstring theory on $\mathrm{AdS}_4 \times {\mathbb {CP}}^3$ with constant dilaton, RR two-form and four-form fluxes. To make the symmetries of the background explicit let us write the metric and the field content in the form

1.15 $$\begin{aligned}&\mathrm{d}s^2_{\mathrm{IIA}}=\frac{R^3}{k}\left( \frac{1}{4} \mathrm{d}s^2_{\mathrm{AdS}_4}+\mathrm{d}s^2_{{\mathbb {CP}}^3}\right) \nonumber \\&\mathrm{d}s^2_{\mathrm{AdS}_4}=R^2(-\mathrm {cosh}^2\rho \mathrm{d}t^2 + \mathrm{d}\rho ^2 + \mathrm {sinh}^2\rho (\mathrm{d}\theta ^2 + \sin ^2\theta \mathrm{d}\phi ^2)) \nonumber \\&\begin{aligned} \mathrm{d}s^2_{{\mathbb {CP}}^3}&=\mathrm{d}\xi ^2+\cos ^2\xi \sin ^2\xi \\&\quad \times \left( \mathrm{d}\psi +\frac{1}{2}\cos \theta _1\mathrm{d}\varphi _1-\frac{1}{2}\cos \theta _2\mathrm{d}\varphi _2\right) ^2 \\&\quad +\frac{1}{4}\cos ^2\xi (\mathrm{d}\theta _1^2+\sin ^2\theta _1 \mathrm{d}\varphi _1^2)\\&\quad +\frac{1}{4}\sin ^2\xi (\mathrm{d}\theta _2^2+\sin ^2\theta _2 \mathrm{d}\varphi _2^2), \\ \end{aligned}\nonumber \\&\begin{aligned} C_1&=\frac{k}{2}((\cos ^2\xi -\sin ^2\xi )\mathrm{d}\psi +\cos ^2\xi \cos \theta _1\mathrm{d}\varphi _1 \\&\quad +\sin ^2\xi \cos \theta _2 \mathrm{d}\varphi _2), \\ \end{aligned}\nonumber \\&\begin{aligned} F_2&=k\Bigg (-\cos \xi \sin \xi \,\mathrm{d}\xi \wedge (2\mathrm{d}\psi +\cos \theta _1\mathrm{d}\varphi _1\\&\quad \quad \qquad -\cos \theta _2\mathrm{d}\varphi _2) -\frac{1}{2}\cos ^2\xi \sin \theta _1\mathrm{d}\theta _1\wedge \mathrm{d}\varphi _1\\&\quad \quad \qquad -\frac{1}{2}\sin ^2\xi \sin \theta _2 \mathrm{d}\theta _2\wedge \mathrm{d}\varphi _2 \Bigg ) \\ \end{aligned}\nonumber \\&F_4=-\frac{3R^3}{8}\omega _{\mathrm{AdS}_4},\quad \mathrm{e}^{2\Phi }=\frac{R^3}{k^3}. \end{aligned}$$

Written in this form, it is easy to see that the background has at least five Killing vectors corresponding to the translations along $t,\psi ,\phi ,\varphi _1,\varphi _2$. The charges associated with the Killing vectors are the energy and the momenta $S,J_1,J_2,J_3$.

To this end it is plausible to make an ansatz for these directions [10]:

1.16 $$\begin{aligned} t=\kappa \tau , \quad \phi =v\tau , \quad \psi =\omega _1\tau , \quad \varphi _1=\omega _2\tau , \quad \varphi _2=\omega _3\tau . \end{aligned}$$

In this setup the authors of [10] found a simple classical string solution and the corresponding charges. The latter are defined as

1.17 $$\begin{aligned} E&=\frac{1}{4}\mathrm {cosh}^2\rho \sqrt{\tilde{\lambda }}\kappa \nonumber \\ S&=\sqrt{\tilde{\lambda }}\int \frac{\mathrm{d}\sigma }{2\pi }v\mathrm {sinh}^2\rho \sin ^2\theta , \nonumber \\ J_1&=\sqrt{\tilde{\lambda }}\int \frac{\mathrm{d}\sigma }{2\pi }\sin ^2\xi \cos ^2\xi \left( \omega _1+\cos \theta _1\frac{\omega _2}{2}-\cos \theta _2\frac{\omega _3}{2} \right) \nonumber \\ J_2&=\sqrt{\tilde{\lambda }}\int \frac{\mathrm{d}\sigma }{2\pi }\left[ \cos ^2\xi \sin ^2\theta _1\frac{\omega _2}{4}+\cos ^2\xi \sin ^2\xi \right. \nonumber \\&\quad \left. \times \left( \cos ^2\theta _1\frac{\omega _2}{4}+\cos \theta _1\left( \frac{\omega _1}{2}-\cos \theta _2\frac{\omega _3}{4}\right) \right) \right] \nonumber \\ J_3&=\sqrt{\tilde{\lambda }}\int \frac{\mathrm{d}\sigma }{2\pi }\left[ \cos ^2\xi \sin ^2\theta _2\frac{\omega _3}{4}+\cos ^2\xi \sin ^2\xi \right. \nonumber \\&\quad \left. \times \left( \sin ^2\theta _2\frac{\omega _3}{4}-\cos \theta _2\left( \frac{\omega _1}{2}+\cos \theta _1\frac{\omega _2}{4}\right) \right) \right] . \end{aligned}$$

The relation between $\tilde{\lambda }$ and the ’t Hooft coupling is $\tilde{\lambda }= \sqrt{32}\pi \lambda$.

If we restrict ourselves to the case of strings moving in $\mathbb {R}_t \times {\mathbb {CP}}^3$, the angular momenta in one $S^2$ are opposite to those in the other $S^2$, while executing motion on $S^1$ in the $U(1)$ Hopf fibration over $S^2 \times S^2$. On the gauge theory side the BPS state corresponding to the string vacuum is $\mathrm {tr}[(A_1B_1)^L]$. If we consider for instance the case of strings with two angular momenta, the corresponding composite states should be $\mathrm {tr}[(A_1B_1)^{J_1}(A_2B_2)^{J_2}]+\mathrm{perm}$.

**A simple semiclassical solution** Below we briefly review the simplest folded string solution of [10]. We will work out this case in detail, while the results for the more complicated solution are given in an appendix. To find a simple folded string solution, in addition to (1.16), let us make the ansatz $\theta _1=\theta _2=0$. The equation of motion for $\xi$ then takes the following form:

1.18 $$\begin{aligned} \xi ''=\frac{1}{4}\sin 4\xi \,\tilde{\omega }^2, \end{aligned}$$

where $\tilde{\omega }=\omega _1+(\omega _2-\omega _3)/2$. The Virasoro constraint is

1.19 $$\begin{aligned} \frac{\kappa ^2}{4}=\xi '^2+\frac{\sin ^2 2\xi }{4}\tilde{\omega }^2. \end{aligned}$$

Because we are looking for a folded string here, $\xi$ will reach its maximal value at some $\xi _0$. At this point $\xi =\xi _0$ we have $\xi '=0$ and therefore $\kappa ^2=\sin ^2 2\xi _0\,\tilde{\omega }^2$. Now the Virasoro constraint has a very simple form:

1.20 $$\begin{aligned} \xi '^2=\frac{\tilde{\omega }^2}{4}(\sin ^2 2\xi _0-\sin ^2 2\xi ), \end{aligned}$$

which is easy to solve in terms of elliptic Jacobi functions. After integration of the above equation from the origin to the turning point we obtain the periodicity condition,

1.21 $$\begin{aligned} 2\pi =4\int _{0}^{\xi _0}\frac{2\mathrm{d}\xi }{\tilde{\omega }\sqrt{\sin ^2 2\xi _0-\sin ^2 2\xi }}. \end{aligned}$$

The angular momenta with this ansatz simply reduce to

1.22 $$\begin{aligned} J_1&= \sqrt{\tilde{\lambda }}\int \frac{\mathrm{d}\sigma }{2\pi }\cos ^2 \xi \sin ^2 \xi \,\tilde{\omega }, \end{aligned}$$

1.23 $$\begin{aligned} J_2&= \frac{J_1}{2} ,\end{aligned}$$

1.24 $$\begin{aligned} J_3&= -J_2. \end{aligned}$$

These relations show that the only independent charges are the energy and one of the angular momenta, say $J_1$. Solving for $\xi$ with the condition (1.21) imposed, we see that the energy and angular momenta of the folded string can be expressed in terms of linear combinations of the complete elliptic integrals of the first kind $\mathbb {K}(k)$ and of the second kind $\mathbb {E}(k)$ with modular parameter $k=\sin 2\xi _0$,

1.25 $$\begin{aligned} E&= \frac{\sqrt{\tilde{\lambda }}}{4}\kappa =\frac{\sqrt{\tilde{\lambda }}}{4}\tilde{\omega }\sin 2\xi _0=\frac{\sqrt{\tilde{\lambda }}}{2\pi }k\mathbb {K}(k), \end{aligned}$$

1.26 $$\begin{aligned} J_1&= \frac{\sqrt{\tilde{\lambda }}}{2\pi }[\mathbb {K}(k)-\mathbb {E}(k)]. \end{aligned}$$

## The dispersion relations

Although there are many interesting developments and applications of this duality, some old questions about the main players in the story are still interesting. For instance, the dispersion relations which are supposed to give the anomalous dimensions are given in implicit form. Most frequently encountered cases are those when the charges are expressed through elliptic integrals which cannot be inverted in closed form. We revisit this problem combining a few approaches to obtain the dispersion relation as series (computable to arbitrary order).

We will approach the problem expanding the expressions for the charges and inverting the series. Expansions for the elliptic integrals in series are given in Appendix A.

Another approach we will use is finding a recurrence for the coefficients using a specific Picard–Fuchs equation. The latter is based on the following simple facts. It is a simple exercise to find that the complete elliptic integrals satisfy the equation

2.1 $$\begin{aligned} \frac{\mathrm{d}\mathbb {K}}{\mathrm{d}k}=\frac{1}{kk^{\prime 2}}(\mathbb {E}-k^{\prime 2}\mathbb {K}), \quad \frac{\mathrm{d}\mathbb {E}}{\mathrm{d}k}= \frac{1}{k}(\mathbb {E}-\mathbb {K}). \end{aligned}$$

After differentiating the above equations we find a special form of the Picard–Fuchs equation,

2.2 $$\begin{aligned} kk^{\prime 2}\frac{\mathrm{d}^2\mathbb {K}}{\mathrm{d}k^2}+(1-3k^2)\frac{\mathrm{d}\mathbb {K}}{\mathrm{d}k}-k\mathbb {K}=0. \end{aligned}$$

The general derivation of the dispersion relations here follows that in [27]. They, however, are derived for spinning strings in the $\mathrm{AdS}_5$ part of the geometry. This means that they describe gauge theory operators with $S$ impurities. We will see later that, surprisingly, the case of rotating strings in the ${\mathbb {CP}}^3$ part of our theory can be manipulated in a very similar way. For completeness, let us give a brief review of the case considered in [27]. The energy and the spin in the case of a spinning string in $\mathrm{AdS}_5$ are

2.3 $$\begin{aligned} {\mathcal E}=\frac{2}{\pi }\frac{k}{1-k^2}\mathbb {E}, \quad {\mathcal S}=\frac{2}{\pi }\left[ \frac{1}{1-k^2}\mathbb {E}-\mathbb {K}\right] . \end{aligned}$$

Applying the above actions, we find

2.4 $$\begin{aligned} \frac{\mathrm{d}{\mathcal E}}{\mathrm{d}k}=\frac{1}{1-k^2}\left[ {\mathcal S}+\frac{1}{k}{\mathcal E}\right] , \quad \frac{\mathrm{d}{\mathcal S}}{\mathrm{d}k}= \frac{1}{1-k^2}[k{\mathcal S}+{\mathcal E}]. \end{aligned}$$

Note that

2.5 $$\begin{aligned} \frac{\mathrm{d}{\mathcal E}}{\mathrm{d}{\mathcal S}}=\frac{1}{k}. \end{aligned}$$

One can proceed with the second derivative

2.6 $$\begin{aligned} \frac{\mathrm{d}^2{\mathcal E}}{\mathrm{d}{\mathcal S}^2}&= \frac{\mathrm{d}}{\mathrm{d}{\mathcal S}}\left( \frac{\mathrm{d}{\mathcal E}}{\mathrm{d}{\mathcal S}}\right) = \left( \frac{\mathrm{d}{\mathcal S}}{\mathrm{d}k}\right) ^{-1}\frac{\mathrm{d}}{\mathrm{d}k}\left( \frac{1}{k}\right) \nonumber \\ \quad&= -\frac{1-k^2}{k^2}\frac{1}{k{\mathcal S}+{\mathcal E}}, \end{aligned}$$

and using (2.5) we find

2.7 $$\begin{aligned} \left( {\mathcal S}+{\mathcal E}({\mathcal S})\frac{\mathrm{d}{\mathcal E}}{\mathrm{d}{\mathcal S}}\right) \frac{\mathrm{d}^2{\mathcal E}}{\mathrm{d}{\mathcal S}^2}+\left( \frac{\mathrm{d}{\mathcal E}}{\mathrm{d}{\mathcal S}}\right) ^3 -\frac{\mathrm{d}{\mathcal E}}{\mathrm{d}{\mathcal S}}=0. \end{aligned}$$

Now one can expand for large ${\mathcal S}$ and find the terms in the expansion. We will apply this approach to our case.

There are two kinds of folded strings—short and long ones. They are distinguished by the modular parameter of the elliptic integrals, small or close to 1, respectively, and therefore the expansion in the two cases is quite different.

Before we start the analysis of the two kind of approximations for the string solutions, namely the short and long folded string, let us make a comment on that. There exists a remarkable duality between the values of conserved charges of the two extreme types of folded strings, that is, short strings ($\sin 2\xi _0\rightarrow 0$) and long strings ($\sin 2\xi _0\rightarrow 1$). The duality is a direct consequence of the Legendre relation that connects complete elliptic integrals of the first and second kind; more precisely

2.8 $$\begin{aligned} \mathbb {E}(k)\mathbb {K}(k')+\mathbb {K}(k)\mathbb {E}(k')+\mathbb {K}(k)\mathbb {K}(k')=\frac{\pi }{2}, \end{aligned}$$

where the complementary elliptic modulus $k'$ is defined as $k^2+k'^2=1$ and $k=\sin 2\xi _0$. By solving (1.25) and (1.26) for $\mathbb {E}(k)$ and $\mathbb {K}(k)$ and substituting in (2.8), we get the following duality relation:

2.9 $$\begin{aligned} \frac{1}{kk'}EE'-\frac{1}{k}EJ_1'-\frac{1}{k'}E'J_1=\frac{\tilde{\lambda }}{8\pi }. \end{aligned}$$

When $k\sim 1$ we have $k'\sim 0$ and vice versa. Equation (2.9) defines a map between energies and angular momenta of short and long strings. Furthermore, it can be rewritten in terms of the anomalous dimensions $\gamma =E-J_1$ as

2.10 $$\begin{aligned} \frac{1}{k'}E'\gamma +\frac{1}{k}E\gamma '+\left( \frac{1}{kk'}-\frac{1}{k}-\frac{1}{k'}\right) EE'=\frac{\tilde{\lambda }}{8\pi }. \end{aligned}$$

**Expansion of the conserved charges for short folded strings** The expansion for the case of short strings is easy to obtain. Here we give the result for completeness. The energy and angular momentum expansions for short folded strings in terms of $k=\sin 2\xi _0$ assume the following forms:

2.11 $$\begin{aligned} E&= \frac{\sqrt{\tilde{\lambda }}}{4}\sum _{n=0}^\infty \left( \frac{(2n-1)!!}{(2n)!!}\right) ^2k^{2n+1} ,\end{aligned}$$

2.12 $$\begin{aligned} J_1&= \frac{\sqrt{\tilde{\lambda }}}{4}\sum _{n=0}^\infty \left( \frac{(2n-1)!!}{(2n)!!}\right) ^2\frac{2n}{2n-1}k^{2n}. \end{aligned}$$

For convenience, we define the following quantities:

2.13 $$\begin{aligned} {\mathcal E}=\frac{2\pi }{\sqrt{\tilde{\lambda }}}E=k\mathbb {K}, \quad {\mathcal J}_1=\frac{2\pi }{\sqrt{\tilde{\lambda }}}J_1=\mathbb {K}(k)-\mathbb {E}(k). \end{aligned}$$

Since the energy and angular momentum represent power series of $x=k^2$, the series (2.12) can easily be inverted either by hand or using a symbolic computational program. Then the inverse spin function $x=x(\mathcal {J}_1)$ may be inserted into (2.11), which leads to the dispersion relation $\mathcal {E}=\mathcal {E}(\mathcal {J}_1)$. The results are

2.14 $$\begin{aligned} x&= \frac{4\mathcal {J}_1}{\pi }-\frac{6\mathcal {J}_1^2}{\pi ^2}+\frac{3\mathcal {J}_1^3}{\pi ^3}+\frac{5\mathcal {J}_1^4}{4\pi ^4}-\frac{9\mathcal {J}_1^5}{16\pi ^5}-\frac{21\mathcal {J}_1^6}{16\pi ^6} \nonumber \\&\quad -\frac{35\mathcal {J}_1^7}{64\pi ^7}+\frac{459\mathcal {J}_1^8}{512\pi ^8}+\frac{5835\mathcal {J}_1^9}{4096\pi ^9}+\cdots \end{aligned}$$

2.15 $$\begin{aligned} \mathcal {E}&= \pi ^{1/2}\mathcal {J}_1^{1/2}+\frac{\mathcal {J}_1^{3/2}}{4\pi ^{1/2}}+\frac{3\mathcal {J}_1^{5/2}}{32\pi ^{3/2}}+\frac{\mathcal {J}_1^{7/2}}{128\pi ^{5/2}} \nonumber \\&\quad -\frac{61\mathcal {J}_1^{9/2}}{2048\pi ^{7/2}}-\frac{201\mathcal {J}_1^{11/2}}{8192\pi ^{9/2}}+\frac{199\mathcal {J}_1^{13/2}}{65536\pi ^{11/2}}+\cdots \nonumber \\ \end{aligned}$$

Going back to the dimensional energy and angular momentum, the latter may also be written in the form

2.16 $$\begin{aligned} E&= \left( \frac{\sqrt{\tilde{\lambda }}J_1}{2}\right) ^{1/2} \left[ 1+\frac{J_1}{2\tilde{\lambda }^{1/2}}+\frac{3J_1^2}{8\tilde{\lambda }}+\frac{J_1^3}{16\tilde{\lambda }^{3/2}}-\frac{61J_1^{4}}{128\tilde{\lambda }^2} \right. \nonumber \\&\quad \left. -\frac{201J_1^{5}}{256\tilde{\lambda }^{5/2}}+\mathcal {O}\left( \frac{J_1^6}{\tilde{\lambda }^3}\right) \right] . \end{aligned}$$

### Expansion of the conserved charges for long folded strings

Now let us try to expand the elliptic integrals and reverse the series following the ideas of [28]. Using formulas (4.8) and (4.10) from Appendix A we can represent the energy and the spin (2.13) in a form suitable for expansion:

2.17 $$\begin{aligned} {\mathcal E}&=\sqrt{1-x}\sum _{n=0}^{\infty }x^n(a_n\ln x+b_n) \end{aligned}$$

2.18 $$\begin{aligned} {\mathcal J}_1&=-1+\sum _{n=0}^{\infty }x^n(a_n\ln x+b_n)+\sum _{n=0}^{\infty }x^{n+1}(g_n\ln x+h_n)\nonumber \\&=-1+b_0+a_0\ln x+\sum _{n=0}^{\infty }x^{n+1}(c_n\ln x+d_n), \end{aligned}$$

where $$x=1-k^2$$ and

2.19 $$\begin{aligned}&a_n=-\frac{1}{2^{2n+1}}\left[ \frac{(2n-1)!!}{n!}\right] ^2,\nonumber \\&b_n=a_n\left[ 2\sum _{k=1}^n\frac{1}{k(2k-1)}-4\ln 2\right] ,\nonumber \\&c_n=a_{n+1}+g_n=\frac{1}{2^{2n+3}}\frac{(2n+1)!!(2n-1)!!}{[(n+1)!]^2}\nonumber \\&\quad =-a_{n+1}\frac{1}{2n+1},\nonumber \\&d_n\!=\!b_{n+1}\!+\!h_n\!=\!c_n\left[ 2\sum _{k=1}^{n+1}\frac{1}{k(2k\!-\!1)}\!+\!\frac{2}{2n\!+\!1}\!-\!4\ln 2\right] . \end{aligned}$$

In (2.18) we separated the term $n=0$ in the first sum and then changed the index as follows: $n\rightarrow n+1$. The explicit values of the first few coefficients are

2.20 $$\begin{aligned}&a_0=-\frac{1}{2},\quad a_1=-\frac{1}{8},\quad a_2=-\frac{9}{128},\nonumber \\&b_0=2\ln 2,\quad b_1=-\frac{1}{4}+\frac{1}{2}\ln 2,\quad b_2=-\frac{21}{128}+\frac{9}{32}\ln 2,\nonumber \\&c_0=-\frac{1}{8},\quad c_1=\frac{3}{128},\quad c_2=\frac{5}{512},\nonumber \\&d_0=\frac{1}{2}-\frac{1}{2}\ln 2,\quad d_1=\frac{9}{128}-\frac{3}{32}\ln 2,\nonumber \\&\quad d_2=\frac{43}{1536}-\frac{5}{128}\ln 2. \end{aligned}$$

In order to reverse the series we represent them in a manner so that they look similar. To accomplish that intention we have to expand the square root and collect the terms in front of $x$ up to $x^2$. Then we shall notice that it is more appropriate not to reverse the series individually one by one but take their linear combination instead. The most convenient linear combination seems to be ${\mathcal E}_1={\mathcal J}_1-{\mathcal E}+1$. After some simple algebra we obtain the following form for ${\mathcal E}_1$:

2.21 $$\begin{aligned} {\mathcal E}_1\!=\!x(a_{10}\ln x\!+\!b_{10})\!+\!x^2(a_{20}\ln x\!+\!b_{20})+{\mathcal O}(x^3), \end{aligned}$$

where

2.22 $$\begin{aligned}&a_{10}=c_0+\frac{a_0}{2}-a_1=0,\nonumber \\&b_{10}=d_0+\frac{b_0}{2}-b_1=\frac{3}{4},\nonumber \\&a_{20}=c_1+\frac{a_1}{2}+\frac{a_0}{8}-a_2=-\frac{1}{32},\nonumber \\&b_{20}=d_1+\frac{b_1}{2}+\frac{b_0}{8}-b_2=\frac{7}{64}+\frac{1}{8}\ln 2. \end{aligned}$$

Reversion of the modified spin function $x=x({\mathcal E}_1)$ passes through the definition of the function $x^*({\mathcal E}_1)$ as a solution of the “reduced” equation

2.23 $$\begin{aligned} {\mathcal E}_1=x^*b_{10}+x^{*2}(a_{20}\ln x^*+b_{20}). \end{aligned}$$

**Fig. 1:**
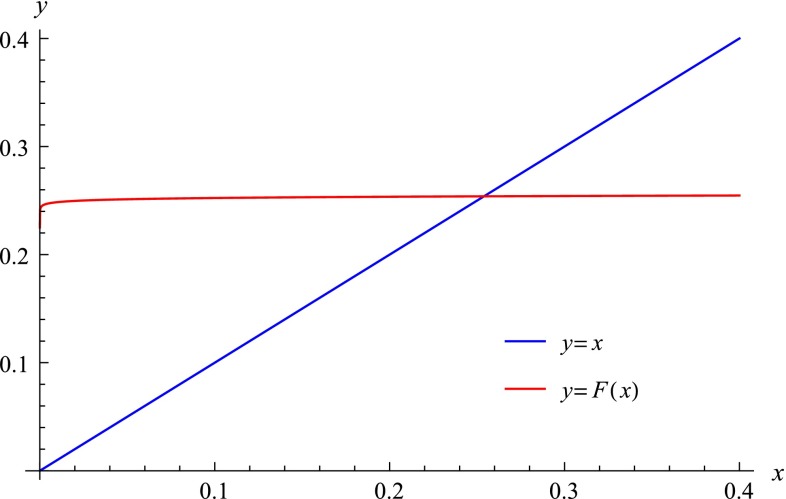
The iteration function $F(x)$

The function $x^*({\mathcal E}_1)$ can be found by iteration of the following map (Fig. 1):

2.24 $$\begin{aligned} F(x)=\frac{{\mathcal E}_1}{b_{10}}-\frac{x^2}{b_{10}}(a_{20}\ln x+b_{20}). \end{aligned}$$

The iteration $x_n=FF\dots F(x_0)$ starts from $x_0={\mathcal E}_1$ and gives

2.25 $$\begin{aligned}&x_0={\mathcal E}_1,\nonumber \\&x_1=\frac{{\mathcal E}_1}{b_{10}}\cdot (1-{\mathcal E}_1A),\nonumber \\&x_2=\frac{{\mathcal E}_1}{b_{10}}\cdot (1-{\mathcal E}_1A)\cdot \left[ 1+\sum _{i=1}^n{\mathcal E}_1^nA^n-\frac{{\mathcal E}_1}{b_{10}^2} \right. \nonumber \\&\quad \left. (1-{\mathcal E}_1A)\left( A-a_{20}\ln b_{10}-\sum _{i=1}^n\frac{{\mathcal E}_1^nA^n}{n}\right) \right] \nonumber \\&\vdots \nonumber \\&x^*, \end{aligned}$$

where $A=a_{20}\ln {\mathcal E}_1+b_{20}$. The above infinite product representation of the iteration procedure is very convenient for computing logarithms of $x^*$. It is clear now that if we want to determine only the unknown constant term and the coefficient in front of $\ln {\mathcal E}_1$, we have to take just the zero iteration $x_0$:

2.26 $$\begin{aligned}&{\mathcal E}\!=\!a_0\ln x\!+\!b_0\!+\!{\mathcal O}(x)\!=\!a_0\ln {\mathcal E}_1\!-\!a_0\ln b_{10}\!+\!b_0\!+\!{\mathcal O}({\mathcal E}_1)\nonumber \\&f=-\frac{1}{2},\quad f_c=b_0-a_0\ln b_{10}=\ln 2+\frac{1}{2}\ln 3. \end{aligned}$$

**Expansion using Picard–Fuchs equations** Let us see now how the idea of using the Picard–Fuchs equations works in our case. Since (2.13) are additive combinations (see Appendix A) of the complete elliptic integrals, they inherit the property that their derivatives with respect to the modular parameter $k$ can be expressed as additive combinations of the complete elliptic integrals and therefore as additive combinations of ${\mathcal E}$ and ${\mathcal J}_1$:

2.27 $$\begin{aligned} \frac{\mathrm{d}{\mathcal E}}{\mathrm{d}k}=\frac{1}{kk'^2}[{\mathcal E}-k{\mathcal J}_1], \quad \frac{\mathrm{d}{\mathcal J}_1}{\mathrm{d}k}=\frac{1}{k'^2}[{\mathcal E}-k{\mathcal J}_1]. \end{aligned}$$

Note the extremely useful property of the derivative

2.28 $$\begin{aligned} \frac{\mathrm{d}{\mathcal E}}{\mathrm{d}{\mathcal J}_1}=\frac{1}{k}. \end{aligned}$$

We need to derive a non-linear differential equation for the function ${\mathcal E}({\mathcal E}_1)$. For this purpose, we find the derivative of ${\mathcal E}_1$ with respect to the modular parameter $k$:

2.29 $$\begin{aligned} \frac{\mathrm{d}{\mathcal E}_1}{\mathrm{d}k}=\frac{\mathrm{d}({\mathcal J}_1-{\mathcal E}+1)}{\mathrm{d}k}=\frac{k-1}{kk'^2}({\mathcal E}-k{\mathcal J}_1), \end{aligned}$$

and notice that the derivative of energy ${\mathcal E}$ with respect to modified spin ${\mathcal E}_1$ again has the property that it depends only on the modular parameter $k$,

2.30 $$\begin{aligned} \frac{\mathrm{d}{\mathcal E}}{\mathrm{d}{\mathcal E}_1}=\frac{1}{k-1}. \end{aligned}$$

Proceeding with the second derivative, we have

2.31 $$\begin{aligned} \frac{\mathrm{d}^2{\mathcal E}}{\mathrm{d}{\mathcal E}_1^2}&=\frac{\mathrm{d}}{\mathrm{d}{\mathcal E}_1}\left( \frac{\mathrm{d}{\mathcal E}}{\mathrm{d}{\mathcal E}_1}\right) =\left( \frac{\mathrm{d}{\mathcal E}_1}{\mathrm{d}k}\right) ^{-1}\frac{\mathrm{d}}{\mathrm{d}k}\left( \frac{1}{k-1}\right) \nonumber \\ {}&=\frac{k(k+1)}{(k-1)^2[{\mathcal E}-k({\mathcal E}_1+{\mathcal E}-1)]}. \end{aligned}$$

Using (2.30) we obtain

2.32 $$\begin{aligned}&\left[ ({\mathcal E}_1-1)\frac{\mathrm{d}{\mathcal E}}{\mathrm{d}{\mathcal E}_1}+{\mathcal E}+{\mathcal E}_1-1\right] \frac{\mathrm{d}^2{\mathcal E}}{\mathrm{d}{\mathcal E}_1^2}+2\left( \frac{\mathrm{d}{\mathcal E}}{\mathrm{d}{\mathcal E}_1}\right) ^3 \nonumber \\&\quad +3\left( \frac{\mathrm{d}{\mathcal E}}{\mathrm{d}{\mathcal E}_1}\right) ^2+\frac{\mathrm{d}{\mathcal E}}{\mathrm{d}{\mathcal E}_1}=0. \end{aligned}$$

Motivated by the type of series ${\mathcal E}({\mathcal E}_1)$ one can make the following ansatz for ${\mathcal E}_1\rightarrow 0$ as a solution of (2.32):

2.33 $$\begin{aligned} {\mathcal E}({\mathcal E}_1)=f_c+f\ln ({\mathcal E}_1)+\sum _{n=1}^{\infty }\sum _{m=0}^{n}f_{nm}{\mathcal E}_1^n\ln ^m{\mathcal E}_1. \end{aligned}$$

This way we obtain recurrence relations between the coefficients

2.34 $$\begin{aligned} f&=-\frac{1}{2},\quad f_{11}=\frac{1}{4},\quad f_{10}=\frac{1}{8}(-3-4f_c), \nonumber \\ f_{22}&=\frac{1}{16},\quad f_{21}=\frac{1}{16}(1-4f_c),\nonumber \\&\quad f_{20}=\frac{1}{64}(1-8f_c+16f_c^2),\nonumber \\ f_{33}&=\frac{1}{24},\quad f_{32}=\frac{1}{32}(3-8f_c),\nonumber \\&\quad f_{31}=\frac{1}{32}(3-12f_c+16f_c^2),\nonumber \\ f_{30}&=\frac{1}{96}(3-18f_c+36f_c^2-32f_c^3),\nonumber \\ f_{44}&=\frac{5}{128},\quad f_{43}=\frac{1}{96}(13-30f_c),\nonumber \\&\quad f_{42}=\frac{1}{256}(53-208f_c+240f_c^2),\nonumber \\ f_{41}&=\frac{1}{256}(39-212f_c+416f_c^2-320f_c^3), \nonumber \\ f_{40}&=\frac{1}{6144}(279-1872f_c+5088f_c^2-6656f_c^3\nonumber \\&\quad +3840f_c^4),\nonumber \\ f_{55}&=\frac{7}{160},\quad f_{54}=\frac{1}{768}(157-336f_c),\nonumber \\&\quad f_{53}=\frac{1}{384}(163-628f_c+672f_c^2),\nonumber \\ f_{52}&=\frac{1}{256}(121-652f_c+1256f_c^2-896f_c^3),\nonumber \\ f_{51}&=\frac{1}{192}(54-363f_c+978f_c^2-1256f_c^3+672f_c^4),\nonumber \\ f_{50}&=\frac{1}{61440}(4347-34560f_c+116160f_c^2\nonumber \\&\quad -208640f_c^3 +200960f_c^4-86016f_c^5), \ldots . \end{aligned}$$

All the coefficients are nicely determined through $f_c$ alone. But we have already determined through the iteration procedure that $f_c=\ln 2+\frac{1}{2}\ln 3$ (cf. (2.26)). Note also that the coefficient $f$ is one and the same in (2.26) and (2.34). Then one can explicitly write down the coefficients (2.34):

2.35 $$\begin{aligned}&f=-\frac{1}{2},\quad f_{11}=\frac{1}{4},\quad f_{10}=-\frac{1}{8}(3+2\ln 12), \nonumber \\&f_{22}\!=\!\frac{1}{16},\quad f_{21}\!=\!\frac{1}{16}(1\!-\!2\ln 12),\quad f_{20}\!=\!\frac{1}{64}(1\!-\!2\ln 12)^2,\nonumber \\&f_{33}=\frac{1}{24},\quad f_{32}=\frac{1}{32}(3-4\ln 12),\nonumber \\&\quad f_{31}=\frac{1}{32}(3-6\ln 12+4\ln ^2 12),\nonumber \\&f_{30}=\frac{1}{96}(3-9\ln 12+9\ln ^2 12-4\ln ^3 12). \end{aligned}$$

### Inverse spin function

In this subsection we start with the expansion using the Picard–Fuchs equation. Next we will proceed with the method suggested in [25].

**Using Picard–Fuchs equations** The non-linear differential equation for the dispersion relation $\mathcal {E}=\mathcal {E}(\mathcal {J}_1)$ is

2.36 $$\begin{aligned} \left( \mathcal {E}(\mathcal {J}_1)\frac{\mathrm{d}\mathcal {E}}{\mathrm{d} \mathcal {J}_1}-\mathcal {J}_1\right) \frac{\mathrm{d}^2\mathcal {E}}{\mathrm{d}\mathcal {J}_1^2}+\left( \frac{\mathrm{d}\mathcal {E}}{\mathrm{d}\mathcal {J}_1}\right) ^3-\frac{\mathrm{d}\mathcal {E}}{\mathrm{d}\mathcal {J}_1}=0. \end{aligned}$$

We will search for a solution by making the following ansatz:

2.37 $$\begin{aligned} \mathcal {E}(\mathcal {J}_1)=\mathcal {J}_1+f_c+\sum _{n=1}^\infty \sum _{m=0}^{n-1}f_{nm}\mathcal {J}_1^m (\mathrm{e}^{-2\mathcal {J}_1-2})^n. \end{aligned}$$

We obtain the following coefficients which all depend on one undetermined coefficient:

2.38 $$\begin{aligned} f_c&=1,\quad f_{21}=f_{10}^2,\quad f_{20}=-\frac{f_{10}^2}{4},\quad f_{32}=2f_{10}^3, \nonumber \\&\quad f_{31}=-\frac{f_{10}^3}{2}, \quad f_{30}=\frac{f_{10}^3}{2},\nonumber \\ f_{43}&=\frac{16f_{10}^4}{3},\quad f_{42}=-f_{10}^4,\quad f_{41}=\frac{19f_{10}^4}{8},\nonumber \\&\quad f_{40}=-\frac{21f_{10}^4}{64}, \nonumber \\ f_{54}&=\frac{50f_{10}^5}{3},\quad f_{53}=-\frac{5f_{10}^5}{3}, \quad f_{52}=\frac{81f_{10}^5}{8}, \nonumber \\&\quad f_{51}=-\frac{55f_{10}^5}{32},\quad f_{50}=\frac{93f_{10}^5}{128}. \end{aligned}$$

This way, we need to obtain the coefficient $f_{10}$ by dint of another independent method. Such a method is introduced in Sect. 2.3 and we will see from (2.63) that $f_{10}=-\frac{1}{16}\cdot 4\cdot (-1)^2\cdot 2^4\cdot \frac{1^0}{1!}=-4$. In addition, all the coefficients (2.38) are in perfect agreement with the ones determined by the method of inverse spin function.

**Using Lambert function** In the rest of this subsection we will follow the method suggested in [25] in order to invert the series of angular momentum $\mathcal {J}_1(x)$, namely $x=x(\mathcal {J}_1)$, and then by substituting $x=x(\mathcal {J}_1)$ into $\mathcal {E}(x)$ to obtain the series of the dispersion relation $\mathcal {E}(\mathcal {J}_1)$ up to some order. For the needs of the upcoming considerations let us write down the series (2.17) and (2.18) in more convenient form. The coefficients we use in this section should not be confused with the coefficients used in Sect. 2.1. We have

2.39 $$\begin{aligned}&\mathcal {E}=\sqrt{1-x}\cdot \sum _{n=0}^\infty x^n(d_n\ln x+h_n)\nonumber \\&\quad =-\sum _{n=0}^\infty x^n\cdot \sum _{k=0}^n\frac{(2k-3)!!}{(2k)!!}(d_{n-k}\ln x+h_{n-k}), \end{aligned}$$

2.40 $$\begin{aligned}&\mathcal {J}_1=\sum _{n=0}^\infty x^n(c_n\ln x+b_n). \end{aligned}$$

Here the series for the energy and angular momentum are written in such a way that they look similar. Each coefficient in (2.39) and (2.40) has the following simple form:

2.41 $$\begin{aligned}&d_n=-\frac{1}{2}\left( \frac{(2n-1)!!}{(2n)!!}\right) ^2, \nonumber \\&\quad h_n=-4d_n\cdot (\ln 2+H_n-H_{2n}), \end{aligned}$$

2.42 $$\begin{aligned}&c_n=-\frac{d_n}{2n-1}, \quad b_n=-4c_n\nonumber \\&\quad \cdot \left[ \ln 2+H_n-H_{2n}+\frac{1}{2(2n-1)}\right] , \end{aligned}$$

where $n=0,1,2,\ldots$ and $H_n$ are the harmonic numbers. We start with solving (2.40) for $\ln x$:

2.43 $$\begin{aligned} \ln x=\left[ \frac{\mathcal {J}_1-b_0}{c_0}-\sum _{n=1}^\infty \frac{b_n}{c_0}x^n\right] \cdot \sum _{n=0}^\infty (-1)^n\left( \sum _{k=1}^\infty c_kx^k\right) ^n. \end{aligned}$$

The above equation looks very complicated but one can get rid of the logarithm by taking an exponent and then (2.43) acquires the following simpler form:

2.44 $$\begin{aligned} x=x_0\cdot \exp {\sum _{n=1}^\infty a_nx^n}=x_0\cdot \exp {(a_1x+a_2x^2+a_3x^3+\cdots )}, \end{aligned}$$

where

2.45 $$\begin{aligned} x_0\equiv \exp \left[ \frac{\mathcal {J}_1-b_0}{c_0}\right] =16\,\mathrm{e}^{-2\mathcal {J}_1-2}. \end{aligned}$$

The advantage of this transformation is that we have an especially convenient series for $x_0$ and the coefficients $a_n$ can easily be determined to the necessary order from (2.43). Note that $x_0$ does not depend on $x$ but only on the angular momentum $\mathcal {J}_1$. This fact allows one to revert the series (2.44) by making use of the Lagrange inversion theorem, thus obtaining the series for the variable $x$ in terms of $x_0$. In our special case one can apply the Lagrange–Bürmann formula in order to get the inverse function:

2.46 $$\begin{aligned} x=\sum _{n=1}^\infty \frac{x_0^n}{n!}\cdot \left\{ \frac{\mathrm{d}^{n-1}}{\mathrm{d}z^{n-1}}\exp \left[ \sum _{m=1}^\infty n\,a_mz^m\right] \right\} _{z=0}. \end{aligned}$$

Differentiating $n$ times and taking the limit $z\rightarrow 0$, we find an explicit form of the inverse function:

2.47 $$\begin{aligned} x=\sum _{n=1}^\infty x_0^n\cdot \sum _{k,j_i=0}^{n-1}\frac{n^k}{n!}\left( {\begin{array}{c}n-1\\ j_1,j_2,\ldots ,j_{n-1}\end{array}}\right) a_1^{j_1}a_2^{j_2}\ldots a_{n-1}^{j_{n-1}},\nonumber \\ \end{aligned}$$

with the following two constraints on the powers of coefficients $a_i$ satisfied:

2.48 $$ \begin{aligned}&j_1+j_2+\cdots +j_{n-1}=k,\quad \& \quad j_1+2j_2\nonumber \\&\quad +\cdots +(n-1)j_{n-1}=n-1. \end{aligned}$$

From (2.43) it can be deduced that all the $a_i$’s are linear in $\mathcal {J}_1$; therefore the inverse spin function $x=x(\mathcal {J}_1)$ necessarily takes the general form

2.49 $$\begin{aligned} x=\sum _{n=1}^\infty x_0^n\cdot \sum _{k=0}^{n-1}a_{nk}\mathcal {J}_1^k, \end{aligned}$$

where the constants $a_{nk}$ have to be determined from (2.47). The fact that the highest degree of $\mathcal {J}_1$ is $k=n-1$ becomes transparent if we combine the two constraints on the values of $j_i$’s as follows:

2.50 $$\begin{aligned} \left. \begin{aligned} j_1+j_2+\cdots +j_{n-1}&=k\\ j_1+2j_2+\cdots +(n-1)j_{n-1}&=n-1 \end{aligned}\right\} \Rightarrow \nonumber \\ k+j_2 +\cdots +(n-2)j_{n-1}=n-1. \end{aligned}$$

Another very important piece of information can be extracted from the constraints (2.50). This information is essential for taking a decision on which of the coefficients $a_i$ make contributions to a certain power of $\mathcal {J}_1$, i.e. which $a_i$ constitute the coefficient $a_{nk}$ for certain $k$. The rule is as follows. The coefficients $a_{n\,n-1}$ (leading terms) are formed by the terms of $a_1$ leading in $\mathcal {J}_1$; the coefficients $a_{n\,n-2}$ (subleading terms) are formed by $a_1$ and the terms of $a_2$ leading in $\mathcal {J}_1$, etc.; thereby the coefficients $a_{n\,n-m}$ are formed by $a_1,\ldots ,a_{m-1}$ and the terms of $a_m$ leading in $\mathcal {J}_1$. This rule becomes obvious if one takes some $j_m\ne 0$ (at least 1), then from $k+j_2+\cdots +(n-2)j_{n-1}=n-1$ it follows that $k=j_m+\cdots +j_{n-1}\le n-m$.

### Anomalous dimensions

Let us now use the obtained general formula (2.47) for $x(\mathcal {J}_1)$ to calculate the anomalous scaling dimensions $\gamma =\mathcal {E}-\mathcal {J}_1$ in the case of spinning in ${\mathbb {CP}}^3$, closed, folded strings as a function of $\mathcal {J}_1$. First we will write down the anomalous dimensions in the already well-known form

2.51 $$\begin{aligned} \mathcal {E}-\mathcal {J}_1=\sum _{n=0}^\infty x^n(f_n\ln x+g_n)=\sum _{n=0}^\infty x^n\left[ A_n+f_n\ln \frac{x}{x_0}\right] , \end{aligned}$$

where the new coefficients are defined by

2.52 $$\begin{aligned}&f_n\equiv -c_n-\sum _{k=0}^n\frac{(2k-3)!!}{(2k)!!}\cdot d_{n-k}, \nonumber \\&\quad g_n\equiv -b_n-\sum _{k=0}^n\frac{(2k-3)!!}{(2k)!!}\cdot h_{n-k},\quad n=0,1,2,\ldots ,\nonumber \\ \end{aligned}$$

and for convenience we introduce $x_0$ by means of the coefficients $A_n$:

2.53 $$\begin{aligned} A_n\equiv g_n+f_n\ln x_0=g_n+2f_n(2\ln 2-\mathcal {J}_1-1). \end{aligned}$$

The expansions of the anomalous scaling dimensions $\gamma =\gamma (\mathcal {J}_1)$ and the inverse spin function $x=x(\mathcal {J}_1)$ contain the same terms, but with different coefficients in front of them:

2.54 $$\begin{aligned}&\text {Leading terms (L): }\mathcal {J}_1^{n-1}(\mathrm{e}^{-2\mathcal {J}_1-2})^n,\nonumber \\&\text {Next-to-Leading/Subleading terms (NL): }\mathcal {J}_1^{n-2}(\mathrm{e}^{-2\mathcal {J}_1-2})^n, \nonumber \\&\text {Next-to-Next-to-Leading terms (NNL): }\mathcal {J}_1^{n-3}(\mathrm{e}^{-2\mathcal {J}_1-2})^n\nonumber \\&\vdots . \end{aligned}$$

The series (2.39), (2.40), and (2.51) look very similar and therefore, if we want to derive $\mathcal {E}-\mathcal {J}_1$ up to a certain order, we need to calculate the inverse spin function $x=x(\mathcal {J}_1)$ up to the same order. This conclusion can easily be seen if we rewrite (2.44) and (2.49) as follows:

2.55 $$\begin{aligned} \ln \frac{x}{x_0}&= \sum _{k=1}^\infty a_kx^k=a_1x+a_2x^2+a_3x^3+\cdots , \end{aligned}$$

2.56 $$\begin{aligned} x&= \sum _{n=1}^\infty x_0^n\cdot \sum _{k=0}^{n-1}a_{nk}\mathcal {J}_1^k=\sum _{n=1}^\infty \mathcal {J}_1^{n-1} x_0^n\cdot \sum _{k=0}^{n-1}\frac{\tilde{a}_{nk}}{\mathcal {J}_1^k} \nonumber \\&= \frac{1}{\mathcal {J}_1}\sum _{n=1}^\infty \mathcal {J}_1^{n}x_0^n\cdot \sum _{k=0}^{n-1}\frac{\tilde{a}_{nk}}{\mathcal {J}_1^k}, \end{aligned}$$

where we redefine the constants $a_{nk}=\tilde{a}_{n\,n-k-1}$ and as before $a_n$ are linear functions of $\mathcal {J}_1$. Finally, the anomalous dimensions (2.51) acquire the following form, convenient for calculations:

2.57 $$\begin{aligned} \mathcal {E}-\mathcal {J}_1&= \sum _{n=0}^\infty x^n\left[ A_n+f_n\ln \frac{x}{x_0}\right] \nonumber \\&= \sum _{n=0}^\infty x^n\left[ A_n+\sum _{k=1}^\infty f_n\,a_kx^k\right] . \end{aligned}$$

**Leading terms** To see how the above considerations work, let us apply them in practice to work out the leading order terms in the large $\mathcal {J}_1$ expansion of anomalous dimensions, which means that we are to find the coefficients of the series

2.58 $$\begin{aligned} E-J_1\bigg |_{\mathrm{(L)}}=\sum _{n=1}^\infty \mathfrak {a}_n\mathcal {J}_1^{n-1}(\mathrm{e}^{-2\mathcal {J}_1-2})^n. \end{aligned}$$

For this purpose, we need to calculate the leading terms of $x$, namely all the constants $\alpha _n$ have to be computed:

2.59 $$\begin{aligned} x_{\mathrm{(L)}}=\sum _{n=1}^\infty \alpha _n\mathcal {J}_1^{n-1}(\mathrm{e}^{-2\mathcal {J}_1-2})^n. \end{aligned}$$

To do so, we have to collect the coefficients that multiply $x^0=1$ on the right-hand side of (2.43) and only coefficients leading in $\mathcal {J}_1$ that multiply $x^1=x$. Thus, (2.43) takes the form

2.60 $$\begin{aligned} \ln x_{\mathrm{(L)}}&= \frac{\mathcal {J}_1-b_0}{c_0}-\frac{c_1}{c_0^2}\mathcal {J}_1\cdot x_{\mathrm{(L)}}\Rightarrow x_0 \nonumber \\&= x_{\mathrm{(L)}} \exp \left[ \frac{c_1}{c_0^2}\mathcal {J}_1\cdot x_{\mathrm{(L)}}\right] =x_{\mathrm{(L)}}\,\mathrm{e}^{\mathcal {J}_1\cdot x_{\mathrm{(L)}}/2}, \end{aligned}$$

where $x_0=16\,\mathrm{e}^{-2\mathcal {J}_1-2}$. We can reverse the above function either by making use of the Lagrange inversion theorem or just by employing (5.6) for the following iterated exponentiation:2.61where we have chosen the principal branch^3^ of the Lambert $W$ function and have derived

2.62 $$\begin{aligned} \alpha _n\equiv (-1)^{n+1}2^{3n+1}\cdot \frac{n^{n-1}}{n!}. \end{aligned}$$

The final step in obtaining the large angular momentum expansion of the anomalous dimensions $E-J_1$ is to put $x_{\mathrm{(L)}}$ into (2.51) and then to retain only terms leading in $\mathcal {J}_1$. Thus we end up with the following series:

2.63 $$\begin{aligned} E-J_1\bigg |_{\mathrm{(L)}}&=\frac{\sqrt{\tilde{\lambda }}}{2\pi }[1+g_1x_{\mathrm{(L)}}-2f_2\mathcal {J}_1x_{\mathrm{(L)}}^2] \nonumber \\&= \frac{\sqrt{\tilde{\lambda }}}{2\pi }\left[ 1-\frac{x_{\mathrm{(L)}}}{4}-\frac{\mathcal {J}_1x_{\mathrm{(L)}}^2}{16}\right] \nonumber \\&=\frac{\sqrt{\tilde{\lambda }}}{2\pi }\Bigg \{1-\frac{1}{4\mathcal {J}_1}[2\,W(8\mathcal {J}_1\, \mathrm{e}^{-2\mathcal {J}_1-2}) \nonumber \\&\quad +W^2(8\mathcal {J}_1\,\mathrm{e}^{-2\mathcal {J}_1-2})]\Bigg \}\nonumber \\&=\frac{\sqrt{\tilde{\lambda }}}{2\pi }\Bigg \{1-\frac{1}{16}\sum _{n=1}^\infty \left[ 4\alpha _n+\sum _{k=1}^{n-1}\alpha _k\alpha _{n-k}\right] \nonumber \\&\quad \cdot \mathcal {J}_1^{n-1}(\mathrm{e}^{-2\mathcal {J}_1-2})^n\Bigg \}. \end{aligned}$$

**Next-to-leading terms** We will sketch in brief the derivation of next-to-leading terms in the large $\mathcal {J}_1$ expansion of anomalous dimensions, i.e. we will calculate the coefficients in the series

2.64 $$\begin{aligned} E-J_1\bigg |_{\mathrm{(NL)}}=\sum _{n=2}^\infty \mathfrak {b}_n\mathcal {J}_1^{n-2}(\mathrm{e}^{-2\mathcal {J}_1-2})^n. \end{aligned}$$

To accomplish that, in addition to the leading terms one should compute also the next-to-leading terms of $x$ in (2.47), more precisely:

2.65 $$\begin{aligned} x_{\mathrm{(NL)}}=\sum _{n=2}^\infty \beta _n\mathcal {J}_1^{n-2}(\mathrm{e}^{-2\mathcal {J}_1-2})^n. \end{aligned}$$

Following again the already familiar strategy, one has to collect the coefficients in front of $x^0$, $x^1$ on the right-hand side of (2.43) and just the coefficients leading in $\mathcal {J}_1$ in front of $x^2$. In consequence (2.43), with accuracy up to subleading order, acquires the form

2.66 $$\begin{aligned} \ln x_{(\mathrm{L} + \mathrm{NL} + \cdots )}&= \frac{\mathcal {J}_1-b_0}{c_0}-\frac{\mathcal {J}_1c_1+b_1c_0-b_0c_1}{c_0^2}\nonumber \\&\quad \times x_{(\mathrm{L}+\mathrm{NL}+\cdots )} \nonumber \\&\quad +\frac{c_1^2-c_0c_2}{c_0^3}\mathcal {J}_1x_{(\mathrm{L} + \mathrm{NL} + \cdots )}^2\nonumber \\&\Rightarrow x_{(\mathrm{L} + \mathrm{NL} +\cdots )}=x_0\exp \nonumber \\&\quad \left[ -\frac{\mathcal {J}_1+1}{2}x_{(\mathrm{L} + \mathrm{NL} + \cdots )}\right. .\nonumber \\&\quad \left. -\frac{7\mathcal {J}_1}{32}x_{(\mathrm{L} + \mathrm{NL} + \cdots )}^2\right] . \end{aligned}$$

The next step is to invert the above equation for $x_{(\mathrm{L}+\mathrm{NL}+\cdots )}$ by making use of the Lagrange–Bürmann formula:

2.67 $$\begin{aligned} x_{(\mathrm{L}+\mathrm{NL}+\cdots )}&= \sum _{n=1}^\infty \frac{x_0^n}{n!}\sum _{\begin{array}{c} k,j_1=0\\ n-1=k+j_1\\ 0\le j_1\le k \end{array}}^{n-1}(-1)^kn^k\frac{(n-1)!}{(k-j_1)!j_1!} \nonumber \\&\quad \times \left( \frac{\mathcal {J}_1+1}{2}\right) ^{k-j_1}\left( \frac{7\mathcal {J}_1}{32}\right) ^{j_1}. \end{aligned}$$

In the above series we need to separate only the subleading $\mathcal {J}_1$-terms, since we have already obtained the leading terms in the previous paragraph. After expansion of the binomial and careful selection of the terms, we find

2.68 $$\begin{aligned} x_{\mathrm{(NL)}}&= \sum _{n=1}^\infty \frac{x_0^n}{n!}\left\{ (-1){n-1}n^{n-1}\frac{n-1}{2^{n-1}} +(-1)^{n-2}n^{n-2} \right. \nonumber \\&\quad \times \left. \frac{7(n-1)(n-2)}{2^{n+2}}\right\} \mathcal {J}_1^{n-2}, \end{aligned}$$

which allows us to write down $x$ up to subleading order in the compact form

2.69 $$\begin{aligned} x_{(\mathrm{L}+\mathrm{NL})}=\sum _{n=1}^\infty (\alpha _n\mathcal {J}_1^{n-1}+\beta _n\mathcal {J}_1^{n-2})\cdot (\mathrm{e}^{-2\mathcal {J}_1-2})^n, \end{aligned}$$

where the $\alpha _n$’s have already been defined by (2.62) and $\beta _n$’s are defined by

2.70 $$\begin{aligned} \beta _n\equiv (-1)^{n+1}2^{3n-2}\frac{n^{n-2}}{n!}(n-1)(n+14). \end{aligned}$$

Now (5.7)–(5.12) can be employed in order to represent the series (2.69) in terms of the Lambert $W$ function with argument $W(8\mathcal {J}_1\mathrm{e}^{-2\mathcal {J}_1-2})$:

2.71 $$\begin{aligned} x_{(\mathrm{L}+\mathrm{NL})}=\frac{2}{\mathcal {J}_1}W-\frac{1}{4\mathcal {J}_1^2}\frac{W^2(7W+8)}{1+W}. \end{aligned}$$

The last thing we have to do is to put (2.71) into (2.51) and retain only the leading and subleading terms. Since we have already obtained the leading terms, here we isolate only the subleading terms which lead us to the final result:

2.72 $$\begin{aligned} E-J_1\bigg |_{\mathrm{(NL)}}\!&=\!-\frac{\sqrt{\tilde{\lambda }}}{128\pi }\sum _{n=1}^\infty \left\{ \!\!\right. 16\beta _n\!+\!\sum _{k=1}^{n-1}\alpha _k[9\alpha _{n-k}\!+\!8\beta _{n-k}]\nonumber \\&\quad \left. +4\sum _{k,m=1}^{n-2}\alpha _k\alpha _m\alpha _{n-k-m}\right\} \cdot \mathcal {J}_1^{n-2}(\mathrm{e}^{-2\mathcal {J}_1-2})^n. \end{aligned}$$

As mentioned above, the results of the three methods are consistent. This means that (2.33), (2.35) and (2.63), (2.72) represent one and the same result, but in two very different forms. The former express the large-spin expansion of the string energy in terms of logarithms of the modified angular momentum $\mathcal {E}_1$ and the latter express the large-spin expansion of anomalous dimensions in terms of the exponent of the angular momentum $\mathcal {J}_1$. It is hard to transform the two types of series into one another. However, one can check their consistency by representing them in power series and compare the coefficients in front of the equal powers. Expressing the anomalous dimensions in different form may be helpful studying different properties of the ABJM theory. We will get back to these issues in a forthcoming publication.

## Conclusions

According to the AdS/CFT correspondence, the anomalous dimensions of the gauge theory operators are given by the dispersion relation of their dual $\mathrm{AdS}_4\times {\mathbb {CP}}^3$ strings. We have computed the large-spin expansion of anomalous dimensions of gauge theory operators in ABJM theory using results from the string theory side. For the simple folded string solutions of [10] the energy and momenta are expressed in terms of elliptic integrals and therefore, to obtain the desired dispersion relations one has to invert the elliptic integrals and solve for the energy in terms of momenta. The inversion of elliptic integrals with respect to the modular parameter $\kappa$ is not an easy task.

We consider two types of folded string solutions, short and long, characterized by the modular parameters of the elliptic functions. Due to the Legendre relation that connects complete elliptic integrals of the first and second kind (2.8), there is a remarkable duality between short and long strings (2.9). According to this formula for each solution of energy $E$ and spin $J_1$, there exists a dual solution whose energy $E'$ and spin $J'_1$ with modular parameters related by $k^2+k'^2=1$. In terms of the anomalous dimensions this duality has the form (2.10). We presented in detail the considerations for the simplest case of folded strings given in [10]. The results for the more complicated case given in that paper contain long and not too informative expressions. They are collected in Appendix C.

We found expressions for the dispersion relations in the form of logarithmic power series and exponential power series in momentum $\mathcal {J}_1$ for leading and first subleading terms (using Lambert functions). We checked that the three approaches we used are consistent giving the same results. It is interesting to note that logarithmic expansions are typical for the expansions in the AdS part of the geometry (see for instance [25]). In the ${\mathbb {CP}}^3$ case the expressions for the energy and the momenta in terms of elliptic integrals are very similar to the $\mathrm{AdS}_5$ case, which shows why this happens in our case.

The results in this note can be extended to include finite size corrections. It would be interesting to pursue the idea to look for recurrent relations allowing one to obtain subleading contributions. Clues for that may come from different approaches we used to obtain the dispersion relations, especially the Picard–Fuchs equation. Promotion of these considerations to the quantum level is also a challenging task.

## Figures and Tables

**Fig. 2:**
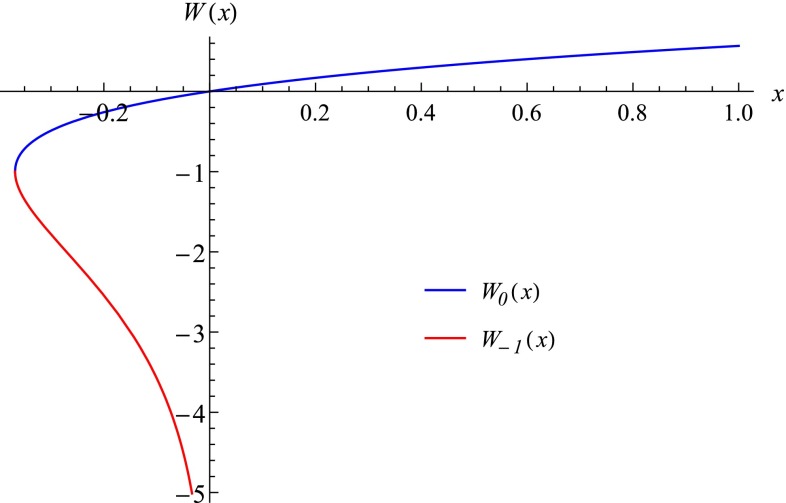
The two real branches of Lambert’s $W$ function

## Appendix A: Elliptic integrals of first and second kind

The defining relations are

4.1 $$\begin{aligned}&\mathbb {K}=\int \limits _0^1\frac{\mathrm{d}x}{\sqrt{(1-x^2)(1-k^2x^2)}} \end{aligned}$$

4.2 $$\begin{aligned}&\mathbb {E}=\int \limits _0^1\frac{\mathrm{d}x\sqrt{1-k^2x^2}}{\sqrt{(1-x^2)}}. \end{aligned}$$

One can pass however, to the Abelian periods known from the algebraic geometry

4.3 $$\begin{aligned} I_1=\int \limits _0^1\frac{\mathrm{d}z}{\sqrt{P(z)}}, \quad I_2=\int \limits _0^1\frac{z\,\mathrm{d}z}{\sqrt{P(z)}}, \end{aligned}$$

where $P(z)=z(1-z)(1-k^2z)$. Then under the identification $z=x^2$ we find the relations

4.4 $$\begin{aligned} \mathbb {K}=\frac{1}{2}I_1, \quad \mathbb {E}=\frac{1}{2}I_1-\frac{k^2}{2}I_2. \end{aligned}$$

The conclusion is that the elliptic functions are additive combinations of the Abelian periods!

One can use the dependence on the modular parameter $k$ ($k^{\prime 2}=1-k^2$) and differentiate the Abelian periods using their defining equations:

4.5 $$\begin{aligned} \frac{\mathrm{d} I_1}{\mathrm{d}k}=\frac{k}{k^\prime }(I_1-I_2), \quad \frac{\mathrm{d} I_2}{\mathrm{d}k}= \frac{1}{kk^{\prime 2}}(I_1-(2-k^2)I_2), \end{aligned}$$

and the corresponding equations for the complete elliptic integrals

4.6 $$\begin{aligned} \frac{\mathrm{d}\mathbb {K}}{\mathrm{d}k}=\frac{1}{kk^{\prime 2}}(\mathbb {E}-k^{\prime 2}\mathbb {K}), \quad \frac{\mathrm{d}\mathbb {E}}{\mathrm{d}k}= \frac{1}{k}(\mathbb {E}-\mathbb {K}). \end{aligned}$$

There is a well-known expansion of the elliptic integral of the first kind $\mathbb {K}$(k) for small values of the module $k^2<1$

4.7 $$\begin{aligned} \mathbb {K}(k)=\frac{\pi }{2}\left\{ 1+\sum _{n=1}^{\infty }\left[ \frac{(2n-1)!!}{(2n)!!}\right] ^2 k^{2n}\right\} , \quad k^2<1 \end{aligned}$$

and for large values $1-k^2<1$

4.8 $$\begin{aligned} \mathbb {K}(k)&=-\frac{1}{2\pi }\sum _{n=0}^{\infty }\left[ \frac{\Gamma (n+1/2)}{n!}\right] ^2(1-k^2)^n\cdot \nonumber \\&\quad \cdot \left[ \ln (1-k^2)+2\psi (n+1/2)-2\psi (n+1)\right] , \end{aligned}$$

where $\psi (z)$ is the digamma function. For the elliptic integral of the second kind $\mathbb {E}(k)$ the expansion for the small values of the module $k^2<1$ is

4.9 $$\begin{aligned} \mathbb {E}(k)= \frac{\pi }{2}\left\{ 1-\sum _{n=1}^{\infty }\left[ \frac{(2n-1)!!}{(2n)!!}\right] ^2 \frac{k^{2n}}{2n-1}\right\} , \quad k^2<1, \end{aligned}$$

and for large values $1-k^2<1$ it is

4.10 $$\begin{aligned} \mathbb {E}(k)&=1\,{-}\,\frac{(1\,{-}\,k^2)}{2\pi }\sum _{n=0}^{\infty }\frac{\Gamma (n\,{+}\,1/2)\Gamma (n\,{+}\,3/2)}{n!(n\,{+}\,1)!}(1\,{-}\,k^2)^n\cdot \nonumber \\&\quad \cdot \,[\ln (1-k^2)+\psi (n+1/2)+\psi (n+3/2)\nonumber \\&\quad -\psi (n+1) -\psi (n+2)]. \end{aligned}$$

## Appendix B: Lambert $W$ function

The Lambert $W$ function is a multivalued function defined as a solution to the following transcendental equation:

5.1 $$\begin{aligned} W(z)\mathrm{e}^{W(z)}=z \end{aligned}$$

for any complex number $z$. $W(x)$ possesses two real branches, explicitly: $W_0(x)$ in $[-\mathrm{e}^{-1},\infty )$ and $W_{-1}(x)$ in $[-\mathrm{e}^{-1},0]$, which are plotted on Fig. 2. The point at which the two branches are connected is $(-\mathrm{e}^{-1},-1)$. Both branches have well-defined Taylor series at $x=0$ [29]:

5.2 $$\begin{aligned} W_0(x)&= \sum _{n=0}^\infty (-1)^{n+1}\frac{(n+1)^n}{(n+1)!}x^{n+1} \nonumber \\&= \sum _{n=1}^\infty (-1)^{n+1}\frac{n^{n-1}}{n!}x^n,\quad |x|\le \mathrm{e}^{-1}, \end{aligned}$$

5.3 $$\begin{aligned} W_{-1}(x)&= \ln |x|-\ln \ln |x|+\sum _{n=0}^\infty \sum _{m=1}^\infty \frac{(-1)^n}{m!}\genfrac[]{0.0pt}{}{n+m}{n+1} \nonumber \\&\quad \times (\ln |x|)^{-n-m}(\ln \ln |x|)^m, \end{aligned}$$

where the unsigned Stirling numbers of the first kind are denoted by $\genfrac[]{0.0pt}{}{n}{k}$. They count the number of permutations of $n$ elements which contain exactly $k$ cycles and can be computed recursively by

5.4 $$\begin{aligned}&\genfrac[]{0.0pt}{}{n}{k}=\genfrac[]{0.0pt}{}{n-1}{k-1}+(n-1)\genfrac[]{0.0pt}{}{n-1}{k}, \nonumber \\&\quad \genfrac[]{0.0pt}{}{n}{0}=\genfrac[]{0.0pt}{}{0}{k}=0, \quad \genfrac[]{0.0pt}{}{0}{0}=1, \quad n,k\ge 1. \end{aligned}$$

The unsigned Stirling numbers of the first kind are related to the harmonic numbers $H_{n-1}$ and generalized harmonic numbers $H_{n-1}^{(2)}$ by

5.5 $$\begin{aligned}&\genfrac[]{0.0pt}{}{n}{1}=(n-1)!,\quad \genfrac[]{0.0pt}{}{n}{2}=(n-1)!H_{n-1},\nonumber \\&\quad \genfrac[]{0.0pt}{}{n}{3}=\frac{1}{2}(n-1)! [H_{n-1}^2-H_{n-1}^{(2)}]. \end{aligned}$$

The W function also gives a solution to the problem of iterated exponentiation . Euler was the first to prove that this iteration converges for real $x$ between $\mathrm{e}^{-e}$ and $\mathrm{e}^{1/e}$ and the limit is

5.6

Using the defining equation (5.1) one can easily find expressions for the derivatives and antiderivatives of Lambert’s function. Here we list some of the most helpful formulas including derivatives and integrals of the $W_0$ function (the so-called principal branch):

5.7 $$\begin{aligned}&W'(x)=\frac{W(x)}{x\left( 1+W(x)\right) } ,\end{aligned}$$

5.8 $$\begin{aligned}&xW'(x)=\sum _{n=1}^\infty (-1)^{n+1}\frac{n^n}{n!}x^n=\frac{W(x)}{1+W(x)} ,\end{aligned}$$

5.9 $$\begin{aligned}&x(xW'(x))'=\sum _{n=1}^\infty (-1)^{n+1}\frac{n^{n+1}}{n!}x^n=\frac{W(x)}{(1+W(x))^3} ,\end{aligned}$$

5.10 $$\begin{aligned}&\int W(x)\,\mathrm{d}x=x\left( W(x)-1+\frac{1}{W(x)}\right) ,\end{aligned}$$

5.11 $$\begin{aligned}&\int \frac{W(x)}{x}\,\mathrm{d}x=\sum _{n=1}^\infty (-1)^{n+1}\frac{n^{n-2}}{n!}x^n=W(x) +\frac{W^2(x)}{2}, \nonumber \\ \end{aligned}$$

5.12 $$\begin{aligned}&\int \frac{1}{x}\int \frac{W(x)}{x}\,\mathrm{d}x^2=\sum _{n=1}^\infty (-1)^{n+1}\frac{n^{n-3}}{n!}x^n \nonumber \\&\quad =W(x)+\frac{3W^2(x)}{4}+\frac{W^3(x)}{6}. \end{aligned}$$

## Appendix C: Folded strings II

There exists another folded string solution [10] that can be obtained if we fix $\xi =\pi /4$. In this case, the only possible way to have a nontrivial solution is to require $\theta _1=\pm \theta _2$. From the equations of motion for $\theta _1$ and $\theta _2$ it follows that this is only possible for $\omega _2=-\omega _3$. Explicitly, the equation of motion for $\theta _1$ has the form

6.1 $$\begin{aligned} \theta _1^{\prime \prime }=\omega _1\omega _2\sin \theta _1, \end{aligned}$$

and it is consistent with the Virasoro constraint

6.2 $$\begin{aligned} \kappa ^2=\theta _1^{\prime 2}+\omega _1^2+\omega _2^2+2\omega _1\omega _2\cos \theta _1. \end{aligned}$$

In the same manner as for the simpler folded string solution, let $\theta _1(0)$ be the maximal value of $\theta _1$. Since $\theta _1$ is a periodic function of $\sigma$, we have

6.3 $$\begin{aligned} -\theta _1(0)\!\le \!\theta _1(\sigma )\le \theta _1(0),\quad \kappa ^2\!=\!\omega _1^2+\omega _2^2+2\omega _1\omega _2\cos \theta _1(0). \end{aligned}$$

There are two different cases here according to the sign of $\omega _1\omega _2$. If $\omega _1\omega _2>0$, $\theta _1$ is varying around $\pi$. On the contrary, if $\omega _1\omega _2<0$, the folded string is centered at $\theta _1=0$. Without loss of generality, we will consider the latter case, i.e. we will assume $\omega _1>0$ and $\omega _2<0$. Now it is easy to integrate the Virasoro constraint,

6.4 $$\begin{aligned} 2\pi =\int _0^{2\pi }\mathrm{d}\sigma =\frac{2}{\sqrt{-\omega _1\omega _2}}\int _0^{\theta _1(0)}\frac{\mathrm{d}\theta _1}{\sqrt{\sin ^2\frac{\theta _1(0)}{2}-\sin ^2\frac{\theta _1}{2}}}. \end{aligned}$$

In this way we get

6.5 $$\begin{aligned} \sqrt{-\omega _1\omega _2}=\frac{2}{\pi }\mathbb {K}(k), \end{aligned}$$

where $k=\sin \frac{\theta _1(0)}{2}$. The energy of the folded string is just $E=\frac{1}{4}\sqrt{\tilde{\lambda }}\kappa$. The angular momenta are

6.6 $$\begin{aligned}&J_1=\frac{\sqrt{\tilde{\lambda }}}{2\pi \sqrt{-\omega _1\omega _2}}((\omega _1-\omega _2)\mathbb {K}(k)+2\omega _2\mathbb {E}(k)), \end{aligned}$$

6.7 $$\begin{aligned}&J_2=\frac{\sqrt{\tilde{\lambda }}}{4\pi \sqrt{-\omega _1\omega _2}}((\omega _2-\omega _1)\mathbb {K}(k)+2\omega _1\mathbb {E}(k)), \end{aligned}$$

6.8 $$\begin{aligned}&J_3=-J_2. \end{aligned}$$

One can show that the following relation is satisfied:

6.9 $$\begin{aligned} \frac{\omega _1}{2}J_1-\omega _2J_2=\frac{\sqrt{\tilde{\lambda }}}{8}(\omega _1^2-\omega _2^2). \end{aligned}$$

In terms of the convenient quantities $\mathcal {E}=\frac{E}{\sqrt{\tilde{\lambda }}},~\mathcal {J}_i=\frac{J_i}{\sqrt{\tilde{\lambda }}},~i=1,2,3$, we have

6.10 $$\begin{aligned}&\omega _1=\mathbb {K}(k)\frac{\mathbb {K}(k)\mathcal {J}_1-2\mathcal {J}_2(2\mathbb {E}(k)-\mathbb {K}(k))}{\mathbb {E}(k)(\mathbb {K}(k)-\mathbb {E}(k))}, \end{aligned}$$

6.11 $$\begin{aligned}&\omega _2=\mathbb {K}(k)\frac{\mathbb {K}(k)\mathcal {J}_2-\mathcal {J}_1(2\mathbb {E}(k)-\mathbb {K}(k))}{\mathbb {E}(k)(\mathbb {K}(k)-\mathbb {E}(k))}, \end{aligned}$$

and the following key relations:

6.12 $$\begin{aligned}&\left( \frac{\mathcal {E}}{\mathbb {K}(k)}\right) ^2-\left( \frac{\mathcal {J}_1+2\mathcal {J}_2}{\mathbb {E}(k)}\right) ^2=\frac{4}{\pi ^2}k^2, \end{aligned}$$

6.13 $$\begin{aligned}&\left( \frac{\mathcal {J}_1-2\mathcal {J}_2}{\mathbb {E}(k)-\mathbb {K}(k)}\right) ^2-\left( \frac{\mathcal {J}_1+2\mathcal {J}_2}{\mathbb {E}(k)}\right) ^2=\frac{4}{\pi ^2}. \end{aligned}$$

The above relations constitute a complicated system in such a manner that it looks hard to exclude the modulus parameter $k$ in order to obtain some kind of dispersion relation. However, using brute force one can derive such a dispersion relation at least for the small values of $k$. To do this, let us introduce new variables, namely $\mathcal {J}_+=\mathcal {J}_1+2\mathcal {J}_2$ and $\mathcal {J}_-=\mathcal {J}_1-2\mathcal {J}_2$. In terms of the new variables (6.12) and (6.13) get the form

6.14 $$\begin{aligned} \mathcal {E}^2&=\left( \frac{4}{\pi ^2}k^2+\frac{\mathcal {J}_+^2}{\mathbb {E}^2(k)}\right) \mathbb {K}^2(k) \end{aligned}$$

6.15 $$\begin{aligned} \mathcal {J}_-^2&=\left( \frac{4}{\pi ^2}+\frac{\mathcal {J}_+^2}{\mathbb {E}^2(k)}\right) (\mathbb {E}(k)-\mathbb {K}(k))^2 . \end{aligned}$$

The main advantage here is that the expansions of the complete elliptic integrals are represented by power series for small values of $k$. It is a laborious task to deal with power series, but still quite straightforward. Hence we will cling to the following procedure. First, we expand the complete elliptic integrals using (4.7) and (4.9). Second, we obtain the power series for $\mathcal {E}$ and $\mathcal {J}_-$ in terms of the variable $x=k^2$. Third, we fix $\mathcal {J}_+$ in (6.15) and reverse the series for $x$. The result for the inverse spin function $x=x(\mathcal {J}_+,\mathcal {J}_-)$ is

6.16 $$\begin{aligned} x&= \frac{2\mathcal {J}_-}{\sqrt{1+\mathcal {J}_+^2}}+\frac{(-3-5\mathcal {J}_+^2)\mathcal {J}_-^2}{2(1+\mathcal {J}_+^2)^2} \nonumber \\&\quad +\frac{(3+15\mathcal {J}_+^2+22\mathcal {J}_+^4)\mathcal {J}_-^3}{8(1+\mathcal {J}_+^2)^{7/2}} +\mathcal {O}(\mathcal {J}_-^4). \end{aligned}$$

Now we have to insert (6.16) in the expansion of (6.14) thus getting a dispersion relation in powers of $\mathcal {J}_-$:

6.17 $$\begin{aligned} \mathcal {E}&= \mathcal {J}_++\frac{\sqrt{1+\mathcal {J}_+^2}\,\mathcal {J}_-}{\mathcal {J}_+}-\frac{(2+3\mathcal {J}_+^2)\mathcal {J}_-^2}{4\mathcal {J}_+^3(1+\mathcal {J}_+^2)} \nonumber \\&\quad +\frac{(8+28\mathcal {J}_+^2+33\mathcal {J}_+^4+ 15\mathcal {J}_+^6)\mathcal {J}_-^3}{16\mathcal {J}_+^5(1+\mathcal {J}_+^2)^{5/2}}+\mathcal {O}(\mathcal {J}_-^4).\nonumber \\ \end{aligned}$$

Finally, one can get back to the original angular momenta $\mathcal {J}_1$ and $\mathcal {J}_2$:

6.18 $$\begin{aligned} \mathcal {E}&=\mathcal {J}_1+2\mathcal {J}_2+\frac{\sqrt{1+(\mathcal {J}_1+2\mathcal {J}_2)^2}\,(\mathcal {J}_1-2\mathcal {J}_2)}{\mathcal {J}_1+2\mathcal {J}_2} \nonumber \\&\quad -\frac{(2+3(\mathcal {J}_1+2\mathcal {J}_2)^2)(\mathcal {J}_1-2\mathcal {J}_2)^2}{4(\mathcal {J}_1+2\mathcal {J}_2)^3(1+(\mathcal {J}_1+2\mathcal {J}_2)^2)}\nonumber \\&\quad +\frac{(8\!+\!28(\mathcal {J}_1\!+\!2\mathcal {J}_2)^2\!+\!33(\mathcal {J}_1\!+\!2\mathcal {J}_2)^4\!+\! 15(\mathcal {J}_1\!+\!2\mathcal {J}_2)^6)(\mathcal {J}_1\!-\!2\mathcal {J}_2)^3}{16(\mathcal {J}_1\!+\!2\mathcal {J}_2)^5(1\!+\!(\mathcal {J}_1\!+\!2\mathcal {J}_2)^2)^{5/2}} \nonumber \\&\quad +\mathcal {O}(\mathcal {J}_-^4). \end{aligned}$$

The AdS/CFT dictionary provides a correspondence between the conserved charges on the string theory side and the composite operators on the gauge theory side. The identification of the operators corresponding to the above angular momenta was suggested in [10],

6.19 $$\begin{aligned} \left\{ \begin{array}{ll} \mathrm{Tr}((A_1B_1)^{\frac{J_{\psi }}{2}+J_{\varphi _1}}(A_2B_2)^{\frac{J_{\psi }}{2}- J_{\varphi _1}}),&{} \text{ for } \omega _1+\omega _2>0, \\ \mathrm{Tr}((B_1^\dagger A_1^\dagger )^{-(\frac{J_{\psi }}{2}+J_{\varphi _1})}(A_2B_2)^{\frac{J_{\psi }}{2}- J_{\varphi _1}}),&{} \text{ for } \omega _1+\omega _2<0. \end{array} \right. \nonumber \\ \end{aligned}$$

Note that, if one takes $\omega _1=-\omega _2$, the above operators reduce to $\mathrm{Tr}(A_2B_2)^{J_{\psi }}$, which is a BPS primary and has $\Delta =J_{\psi }$ without quantum correction, which is in disagreement with the string calculation. Since the dispersion relations (6.12) and (6.13) are the same as in the $\mathrm{AdS}_5\times S^5$ case, it is natural to accept that point of view, namely, that there exists an interpolating function [30] governing the anomalous dimension transition from $g^2$ in the weak coupling regime [13–17] to $g$ in the strong coupling regime [18, 31] .

## Footnotes

1 See also [8].

2 There are huge amount of papers on ABJM theory. Here we will quote only those which are directly related to our study and which we really used.

3 The principal branch provides the correct limiting behavior of $x$, i.e. $x\rightarrow 0^+$ when $\mathcal {J}_1\rightarrow +\infty$.
